# Integrating mobile-phone based assessment for psychosis into people’s everyday lives and clinical care: a qualitative study

**DOI:** 10.1186/1471-244X-13-34

**Published:** 2013-01-23

**Authors:** Jasper E Palmier-Claus, Anne Rogers, John Ainsworth, Matt Machin, Christine Barrowclough, Louise Laverty, Emma Barkus, Shitij Kapur, Til Wykes, Shôn W Lewis

**Affiliations:** 1 Division of Clinical Psychology, School of Psychological Sciences, the University of Manchester, Oxford Road, Manchester, United Kingdom; 2 Faculty of Health Sciences, University of Southampton, Highfield, Southampton, United Kingdom; 3 Institute of Population Health, the University of Manchester, Oxford Road, Manchester, United Kingdom; 4 Department of Sociology, Social Policy and Criminology, the University of Liverpool, Liverpool, United Kingdom; 5 School of Psychology, University of Wollongong, Northfields Avenue, Wollongong, Australia; 6 Institute of Psychiatry, Kings College London, London, United Kingdom; 7 Institute of Brain, Behaviour and Mental Health, the University of Manchester, Oxford Road, Manchester, United Kingdom; 8 Room 3.309, Jean McFarlane Building Community Based Medicine, The University of Manchester, Oxford Road, Manchester, M139PL, UK

**Keywords:** Mobile-phone, Psychosis, Assessment, Ambulant, Schizophrenia

## Abstract

**Background:**

Over the past decade policy makers have emphasised the importance of healthcare technology in the management of long-term conditions. Mobile-phone based assessment may be one method of facilitating clinically- and cost-effective intervention, and increasing the autonomy and independence of service users. Recently, text-message and smartphone interfaces have been developed for the real-time assessment of symptoms in individuals with schizophrenia. Little is currently understood about patients’ perceptions of these systems, and how they might be implemented into their everyday routine and clinical care.

**Method:**

24 community based individuals with non-affective psychosis completed a randomised repeated-measure cross-over design study, where they filled in self-report questions about their symptoms via text-messages on their own phone, or via a purpose designed software application for Android smartphones, for six days. Qualitative interviews were conducted in order to explore participants’ perceptions and experiences of the devices, and thematic analysis was used to analyse the data.

**Results:**

Three themes emerged from the data: i) the appeal of usability and familiarity, ii) acceptability, validity and integration into domestic routines, and iii) perceived impact on clinical care. Although participants generally found the technology non-stigmatising and well integrated into their everyday activities, the repetitiveness of the questions was identified as a likely barrier to long-term adoption. Potential benefits to the quality of care received were seen in terms of assisting clinicians, faster and more efficient data exchange, and aiding patient-clinician communication. However, patients often failed to see the relevance of the systems to their personal situations, and emphasised the threat to the person centred element of their care.

**Conclusions:**

The feedback presented in this paper suggests that patients are conscious of the benefits that mobile-phone based assessment could bring to clinical care, and that the technology can be successfully integrated into everyday routine. However, it also suggests that it is important to demonstrate to patients the personal, as well as theoretical, benefits of the technology. In the future it will be important to establish whether clinical practitioners are able to use this technology as part of a personalised mental health regime.

## Background

Policy makers in the United Kingdom, and elsewhere, have increasingly advocated the use of mobile-phones in the treatment of patients with long-term conditions, based on the presumption that they have the capability to improve both clinical care and self-management [1]. However, in the field of mental health, little is currently understood about the acceptability of new technological systems or their integration (Daker White G, Rogers A: What is the potential for social networks and support to enhance future telehealth interventions for people with a diagnosis of schizophrenia? A critical interpretive synthesis. Submitted). In particular, the perception of mobile-phone based interventions in individuals with diagnoses of severe mental illness has not previously been sought. This paper outlines a qualitative evaluation of mobile-phone based assessment in patients with schizophrenia and related disorders.

Schizophrenia can be distressing and disabling with many individuals experiencing an episodic, lifelong course of fluctuating symptoms [2]. Individuals for the most part live and manage their conditions in the community and there can be lengthy and inconsistent periods of time between clinical assessments. This may mean that symptom deterioration and relapse indicators are sometimes missed, and opportunities for promoting self-management lost. Mobile-phone based assessment can upload data in real-time, helping to facilitate earlier and more effective intervention. It could also be used to promote greater self-monitoring strategies increasing opportunities to directly modify behaviour and engage in informal support [3]. The flow and frequency of reports back about a persons’ condition could in turn enhance the potential for therapeutic alliance through better considered and informed decision making with clinicians, and increased levels of perceived control, autonomy and self-esteem in service users. Service user feedback advocates autonomy and feelings of control as key components of subjective recovery from severe mental illness [4]. Additionally, a greater understanding of the self, empowerment, and collaborative care have been identified as factors influencing recovery from psychosis [5]. Thus, the aims and utility of healthcare technology appear compatible with service user definitions of recovery. The extent to which this is likely to happen in practice is part of the rationale for carrying out the study reported in this article.

There are multiple technological options for delivering mobile-phone based assessment. Smartphone software applications can be purpose-made allowing for greater configurability and usability [6]. Alternatively, text messages have the major advantage that they are not constrained by the make or model of the user’s mobile phone [7]. Mobile-phone technology is becoming increasingly widespread and affordable in individuals with mental illness, with one report showing that rates of use are similar to those of the general population [8,9]. With regards to their effectiveness, small-scale text-based interventions in psychotic samples have shown a significant reduction in the severity of hallucinations [10] and the number of acute inpatient admissions [11]. Our research group has demonstrated high compliance and low drop-out rates to smartphone facilitated assessment in individuals with psychotic experiences [12]. Analysis of quantitative feedback data also revealed that whilst smartphones outperform text based solutions in terms of data-point completion and length of entry times, patients’ appraisals of both forms of technology are generally positive [13].

Early evaluations of mobile-phone based assessment attest to its efficacy. However, the question of which technology and how it is implemented are always more complex issues than is originally anticipated [14]. Assumptions about an objects use and how patients’ engage with technology in reality may not match preconceived ideas. Indeed, large trials of mobile interventions in physical disorders report high levels of non-participation and withdrawal suggesting that these technologies are often poorly integrated into clinical practice [15-17]. Additionally, these problems appear to occur at all stages of implementation and integration, including at a service level [18]. The complexities of a technology’s expected value versus its actual use in practice, especially with regards to social relations, make a focus on the processes between users and context essential. These questions might be best addressed through qualitative design research [19].

Qualitative studies, in the realm of physical disease management, have suggested that patients can be ambivalent about healthcare technology, views mediated by the technologies place of use (i.e. home vs. public environments) [20]. Implementation is often obstructed by system design; uneven integration resulting from poor information transmission concerning the role of technological systems and their adoption; contextual relevancy; and uncertainties about novel software’s adequacy and purpose [21]. Furthermore, threats to independence, prior identities and established ways of self-managing, perceived technical competence, and how technology disrupts healthcare practices, were cited as reasons for non-participation or withdrawal from a recent trial for mobile-phone based intervention in the UK [22]. Healthcare technologies have been found to positively impact on patients' sense of autonomy and participation in social activities, but can also greatly restrict behaviour [20]. For example, in regards to the latter, technology that draws attention to an individual may make them feel uneasy and avoid public spaces.

Although single studies can be revealing, reviews allow new insights and reinterpretations of themes from across different clinical groups and software packages [23]. A recent meta-synthesis identified several factors influencing how technology is integrated into the management of chronic conditions, including preventing individuals from recreating a normalized identity, the generation of new uncertainties, issues with technological dependence, problems with adapting to a technologically assisted life, and difficulties with actually using the devices [24]. In analysis of 16 studies, Obstfelder and colleagues [25] observed that successfully implemented medical software applications had clear benefits to the challenges faced by local services, and provided solutions to medical and political issues (e.g. targeting individuals in inaccessible geographical regions). A collaborative enterprise between promoters and users, and the presence of a clear organisational and technical arrangement within a service, were found to further facilitate successful adoption of technology. Thus, a multitude of factors may influence the accommodation of mobile health devices into domestic settings, and its ability to make the transition from the foreground to the background of peoples’ lives [26]. Integration and acceptance may be vital in determining mobile-phone technologies ability to assist with long-term disease management.

The aim of this study was to explore patients’ understandings and perceptions of mobile-phone based clinical assessment for psychosis, and how it might be implemented into their everyday lives and clinical care. The contents represent the qualitative component of a mixed-methodology evaluation of both text and native Smartphone application administered assessments for psychosis. The quantitative part of the study explored differences in the acceptability and feasibility of the two forms of software, and can be found in a separate manuscript [13]. To the best of the author’s knowledge a qualitative exploration of mobile healthcare technologies has never previously been conducted in individuals with schizophrenia (Daker White G, Rogers A: What is the potential for social networks and support to enhance future telehealth interventions for people with a diagnosis of schizophrenia? A critical interpretive synthesis. Submitted).

## Methods

### Participants

The study received approval from the North West One National Health Service Research Ethics Committee (ref: 11/H1017/3) and all participants provided informed consent to take part. Data was collected as part of an evaluation of mobile-phone based assessment methods in community dwelling individuals meeting or having met the criteria for a Diagnostic and Statistical Manual (Fourth Edition; DSM-IV) diagnosis of non-affective psychosis. Participants were required to currently own and have access to a mobile phone for the purpose of the study. Inpatient status, and organic or substance induced psychosis were exclusion criteria.

38 individuals were referred to the research team to take part in the study. Of these eight changed their minds, three couldn’t be contacted, and three were deemed ineligible prior to consent. This provided a final sample 24 individuals who provided informed consent. All consenting participants completed the qualitative interview at the end of the study, although the number of completed data-points varied considerably between individuals. One participant requested that the text-based system be turned off two days early as it was making her preoccupied with her thoughts. The demographic and clinical information for the sample is provided in Table 1.

**Table 1:** **Demographic and clinical information for sample (*n*=24)**

	
**Age, mean (*SD*)**	**33.04 (9.5)**
Inpatient admissions, mean *(SD)*	2.3 (2.5)
Males, *n*	19
Diagnosis, *n*	
Schizophrenia	22
Schizoaffective disorder	2
Medication, *n*	
Antipsychotic	20
Mood stabiliser	2
Medication free	2
Ethnicity, *n*	
White British	17
Mixed-Race British	2
Black British	2
Black African	1
Black Carribean	1
White Other	1
Service recruited through, *n*	
Community Mental Health Team	15
Early Intervention Team	8
Supported living staff	1

### Equipment

The native Smartphone application was specifically developed for Android Smartphones. Android is an operating system created by Google which runs on mobile phones from different manufacturers. Although this software was developed to run on any Android phone for this study we used the Orange San Francisco device, as has recently been outlined in other publications [12]. For the purpose of this study, the software application was not wirelessly enabled and all answers were stored on the mobile-phone handset for downloading at the end of the sampling procedure.

The text-messages (SMS) operated from openCDMS, a secure online platform, which facilitated both the sending of questions and the storing of responses [27]. Each text contained an individual question to which the participant replied with a number from 1 (‘disagree’) to 7 (‘agree’). Therefore, each individual received multiple text messages, one for each question within a set. Although multiple questions within a single text message was also a feasible option it was thought that this would be a more confusing and error prone process.

### Design

A randomised repeated measures cross over design study was employed, where all participants completed six days of assessment with each form of technology (SMS or Smartphone software application), with a 7-day rest period in between. For assessment with the software application participants were given a University of Manchester owned Smartphone for the period of sampling. The text message system was conducted on participants own mobile-phones. In order to reduce burden on participants there were two alternating sets of questions, which were assessed at four time-points each day for the duration of sampling. Items were branched so that later questions were contingent on an individual’s earlier responses [12]. For example, if an individual endorsed the item ‘I have felt worried, nervous or anxious’ (i.e. scored >2 out of 7) then they were also presented with the question ‘my anxiety has stopped me from doing things’. Question sets were created to measure a range of psychotic and related symptoms. These were hopelessness (2–4 items; e.g. ‘I have felt like the future holds little for me’), depression (2–6 items; e.g. ‘I have felt sad’) and hallucinations (2–8 items; e.g. ‘I have heard voices’) in set one, and anxiety (1–4 items; e.g. ‘My anxiety has stopped me from doing things’), grandiosity (2–3 items; e.g. ‘I have felt like I have powers or abilities that other people don’t have’), paranoia (3–6 items; e.g. ‘I have been suspicious’) and delusions (0–8 items; e.g. ‘I have felt like I could read other people’s thoughts’) in set two.

Qualitative interviews were undertaken as part of a debriefing session at the end of the second period of sampling, which lasted approximately 20 minutes to 1 hour. Quantitative feedback data was also collected in order to compare text and smartphone assessment methods, but this is presented in a separate manuscript [13].

### Qualitative interviews

A semi-structured interview schedule was developed to explore people’s understanding and perceptions of mobile-phone based clinical assessment for psychosis, and how it might be implemented into their everyday lives and clinical care. Summaries of the emergent themes are provided in Tables 2, 3 and 4.

**Table 2:** Quotes for the first theme – The appeal of usability and familiarity

	
Speed of entry	I do think it is good for that reason that it’s very easy, it pops up and tells you when you need to do it and all you need to do is just slide it and it seems quicker as well. With the text it was like more thinking between the numbers. You had to actually spend more time thinking about it. ID9 (male in his late teens)
	It were a lot faster to fill in. The questions came up straight away… I’d say it probably took three times longer by text than on the Smartphone. ID18 (male in his twenties)
	But your phone it was just on and it was just … on the bar you just done it easy. Speedy and all that. I’d rather your phone than my phone, just for that though. ID19 (male in his forties)
Technological issues	Also depended on having a signal, so I couldn’t send text messages if there was no signal. ID3 (male in his twenties)
	You see that phone, I’ve only charged it twice. And it’s still got pretty much full battery power. That’s… pretty good battery life on that. I mean, quite resilient them ones. I wish my battery were like that on my phone. ID8 (male in his late teens)
	Just deleting it was …it was a pain. Went through it and then find delete and then doing it and reading the next question and doing it again and deleting it, it was just doing it all over and over again. ID19 (male in his forties)
Familiarity and ease of transport	I don’t know, because I am familiar with the buttons and all that with my phone, know what I mean, what to press and what not to and how to answer it, answer the questions and numbers and buttons to press. I know which ones on my phone. ID5 (male in his forties)
	With the text you don’t have to learn how to use anything… So like maybe for older people you know, they’re less likely to want to do it and… I think the Smartphone are a lot better overall though. ID18 (male in his twenties)
	It was just a bit of bother having 2 phones like to carry around, have my normal one and one for the testing but if that was going to be testing on someone’s normal phone as like an integrated App for them then it's not going to be a problem is it. ID15 (male in his twenties)

**Table 3:** Quotes for the second theme - Acceptability, validity, and integration into domestic routines

	
Repetition and boredom effects	I don’t want to say a bad thing but…I thought there would be a variety of different questions and all that lot, which there is not though. It's just sort of like 2 or 3 questions and it asks you over and over again. ID5 (male in his forties)
	In the interviews they’re different. But in the, they’re different questions in the interview so in a way I do like the interview because it’s like something different but there’s just the same questions all the time in… in the phone. It’s like constantly the same questions they’re asking. Like in the interview it varies a bit. I’m not sure I do prefer, I don’t know which one I prefer. ID9 (male in his late teens)
	I think people would become tired of the same questions, need different questions. ID14 (male in his forties)
Validity of the questions	Someone who is more suspicious might just throw away your phone and you’ve lost like loads of info or you get someone who just lies on it and you think wow this persons getting better and they are not, this is like quantitative stuff isn’t it so as long as it’s like balanced with interviews however often that person needs yeah but I wouldn’t give all the power to the robots just yet… I just think it would be useful but not to put all eggs in one basket. ID3 (male in his twenties)
	I think you need more questions to know about how the person is actually feeling… Because certain, like, why are you feeling sad for instance and stuff like that, and like put more into the questions, like say you’re saying I’m feeling sad, then put why are you feeling sad. For example. ID11 (female in her forties)
	I am not sure it captures what you are experiencing but yeah I mean…I don’t know. ID21 (male in his thirties)
Biographical disruption & symptomatic reactivity	I have a terrible memory which is good in certain situations so forget the bad stuff which helps, but this kicks my memory up so makes me appreciative of when am feeling good. ID3 (male in his twenties)
	Yes I wouldn’t say the way the questions … they are a little bit uncomfortable at times but most of them are fine. Just the self harm one really and you know when it said did this make you sad, you think well actually it did and realise … you don’t get any sadder but you just realise you’re not where you are. ID18 (male in his twenties)
	INT Did it make you feel worse or better? R A little bit worse at times. Yes. INT Worse in what ways? R Remembering how ill I was. ID24 (male in his forties)
Ease of integration	Didn’t stop me from doing anything no cause if I was at Uni I wouldn’t have my phone with me anyway. No. I mean sometimes I would be watching the TV or playing a game and the questions arrived and I just paused for 5 minutes answered the questions and then resume. ID4 (male in his twenties)
	I had to go to the gym on Monday, I noticed that if I wanted the gym I might have missed again because I remember when I had this Smart phone I went to the gym on Monday and I missed it…I missed filling the question because when I came back to take my stuff I saw that you have run out of time to fill in the questionnaire. But this time around I didn’t go to the gym just because of that; I didn’t want to miss it. ID17 (male in his thirties)
	Like I’ve got loads of chemist, Monday and Friday … Monday, Wednesday and Fridays but I didn’t want to get half way there and the questions starting coming up so I kind of waited until the first set of questions come on then you know then you’ve like half an hour to an hour’s difference to get there and back. You know what I mean. You just didn’t want them to catch you out you know, like half way there and there’s be this beep going. ID19 (male in his forties)

**Table 4:** Quotes for the third theme – Perceived impact on clinical care

	
More detailed information	It can only be beneficial to them [clinical team]. As far as I can see. Knowing what mood you are in during the day, and different times of the day and when people are out there it can only be beneficial to them. ID20 (male in his forties)
	You can get many answers. That’s what I think. You can get many answers and many questions can be asked and stuff like that. From all that one bit of knowledge that you have from what people have filled in. You know, just them questions. ID6 (male in his twenties)
	If you had this for 3 months say standard, you would get a very intense care kind of thing and you could direct care more effectively, I think if you could see they are more at risk and head it off at the pass sort of thing. ID3 (male in his twenties)
Early intervention	Well its useful, you want to know if, someone like myself or others who are suffering from mental illness, you want to know if they’re in that zone where they’re bad enough to like you say harm themselves, you want to get that gist do you know whatever. Or maybe whether they’re going to violent towards others, I don’t know. It might work that way, yeah. I think in those ways it could be helpful, in that way. That’s the way I saw it. ID10 (male in his thirties)
	It can nip it in the bud before it gets any worse. ID11 (female in her forties)
	Well people can be monitored quickly with the response and they can work out what the people’s needs are on a particular day, if they need to. ID24 (male in his forties)
Benefits to communication	I think your ways easier. I think it’s a lot better if it’s put on the phone because that makes it not out loud and stuff… That’s why I’ve done it. Because it’s not out loud, it’s on a screen. It’s a lot easier. ID7 (female in her twenties)
	I mean it's harder for a person to sum up a week of symptoms and experiences in sort of however long the interview takes anyway. People’s memories get clouded and sort of however you are feeling at that moment sort of bias what you are saying to the interviewer anyway. So I mean if you are having something that tests your regular interview or regular intervals and is purely detached, it's passionate, then you are going to get more valuable data than something that can be woolly and rambling, if you know what I mean. ID15 (male in his twenties)
	When I hear voices it’s dependant a lot on my situation… how I feel. Like that makes it worse and better and they’re [clinicians] asking me to quantify how much better it’s got but the problem is that it goes week by week so you can’t quantify it over a month. You know some weeks I’d had a fine week and not heard any at all and some weeks I’d had like 5 days in a row where I’d heard them and he wanted to … he [psychiatrist] was saying you know, quantify it and you just … like for me, I can’t and I almost end up arguing with him over it. ID18 (male in his twenties)
Threat to existing care	Yeah the person can be from your Team for instance. Would be ready to listen because some people take interest in listening…I mean some people like it when they have listened, they are being listened you know. They can say what they want. Whatever is in their belly, they just want to lay it out you know. ID17 (male in his thirties)
	Well its contact isn’t it, its personal contact. You know you, it's much better to have somebody there in person. That’s what I think anyway. ID20 (male in his forties)
	So if I was really distressed and I believed everything my brain was telling me I would want to speak to a Psychiatrist. ID23 (male in his forties)
Relevancy of intervention	Well I see the staff every day anyway so…it probably wouldn't make much difference really. ID2 (male in his thirties)
	I don't tell anybody because to me it's not an issue anymore it's in the past now so if I want to move on I better forget everything in the past, that was in the past and move on so it's not relevant for me to mention again my mental issue so something like that…because I don’t consider me to be mentally ill anymore. ID17 (male in his thirties)
	People who have been unwell and are getting better then it's not going to be as useful is it. I mean they probably would see it as being a waste of time them doing this. ID21 (male in his thirties)

The interview schedule covered: i) an evaluation of the technology; ii) an evaluation of the assessment procedure; iii) integration into, impact on and disruption to everyday routine and activities; iv) acceptability and reasons for non-compliance; v) existing healthcare protocols and perceived impact on clinical care and treatment; and vi) long-term implementation. Although most questions related to the individual’s current situation, they were also asked to explore these topics in relation to periods of acute illness, and other people’s expected involvement in and reactions to the technology.

### Data analysis

The qualitative interviews were recorded on a portable Dictaphone device, and were transcribed verbatim. A framework analysis was used to identify themes and sub-themes, which were coded in thematic charts [28]. AR, LL and JPC examined transcripts independently and met to discuss emerging codes. Themes identified in the early interviews were used to identify areas of investigation in the later interviews. Thus, the themes underwent several iterations. The presented quotes were collected from across the sample of participants, as representative examples of the three final themes.

## Results

Three themes relating to the mobile-phone technology emerged from the analysis of the data. These were ‘the appeal of usability and familiarity’, ‘acceptability, validity and integration into domestic routines’, and ‘perceived impact on clinical care’. Further examples of quotes for each of the themes are provided in Tables 2, 3 and 4.

### Theme 1. The appeal of usability and familiarity

Participants were asked about their preferences for different forms of technology (native software application or text messages). Opinions varied according to whether the user identified a preference for the more streamlined and technologically advanced native Smartphone software application, or the security and awareness of their own phone. Participants often identified that the software application was fast and easy to use, causing little disruption to everyday activities. By comparison, the process of sending multiple text messages was seen as awkward, laborious and burdensome. There was a common expectation that modern technology should be user-friendly and hassle free, which was deemed to be vital when implementing mobile-phone assessment as a long-term monitoring strategy.

‘Cause that was simple because you can just (making swoosh noises) answer straight away, it was easy to answer but on my phone I have to reply, press reply and then select 1 and then send it, wait again, press again, reply and select and so on. Yeah so that was the difference.’ ID17 (male in his thirties)

The usability of the two devices was also dictated by the technological difficulties that participants’ encountered. They were sometimes critical of their own (occasionally old) phones for having poor battery life, repeated text alerts, and limited space for new messages. Some individuals noted poor signal as a reason for missing data. A common perceived limitation of employing the native software application, especially in those unfamiliar with Smartphones, was the use of the touch-screen, which many participants found unresponsive and difficult to adjust to. However, although some individuals found it difficult to adapt to the more modernised touch-screen analogue scale, others stated that they found it more straightforward and easier to use than text messages.

‘I’d say it was quite quick. It was quite easy to use, but sometimes the worst thing was I had to touch it quite a few times on occasions for it to work properly which was a bit frustrating. On one occasion when it happened it was difficult to get it accurately because my finger went to slightly the wrong place on the scale.’ ID14 (male in his forties)

The utility of the text-based system was also raised. Participant’s often discussed feeling more comfortable and secure with a device with which they were familiar, rather than new and unknown technology. Some individuals stated that they were wary about pressing the wrong buttons on the Smartphone, which would have led to them being unable to remove themselves from complicated software applications. Participants also expressed their displeasure at having to carry around two mobile-phone handsets, stating that it would be more convenient to just use their own phone.

'You know so there is not much difference between the two I don’t think. I only preferred my phone because I have had it longer and I am more used to it.’ ID2 (male in his thirties)

‘I liked that but because it was on my own phone. I could just do it wherever and I’d always have my phone with me and it was easier for that, but like I say if I did have the app on this phone I do think that would be better yeah.’ ID9 (male in his late teens)

### Theme 2: Acceptability, validity, and integration into domestic routines

Participants discussed the acceptability of the mobile-phone assessment procedure, and how they integrated it into their daily routine. Many individuals felt that although the procedure was acceptable over the two weeks of sampling, they would be reluctant to complete questions over longer periods of time. A large number of people noted that the repetitiveness of the questions led to disinterest, which would only increase if sampling was prolonged. Although participants recognised the advantages of intense monitoring in clinical assessment, many emphasised the need for variation in the content, number and order of items in order to avoid boredom and fatigue effects. Thus, the perceived personal value of this technology was not considered to be strong enough to motivate long-term adoption.

‘It’s alright but a bit repeated. You know, kept on going over and over and over the same things. There wasn’t anything new’. ID19 (male in his forties)

Participants also talked about the extent to which their mood, symptoms and thoughts changed in response to completing mobile-phone assessment (i.e. reactivity to the methodology). A number of patients noted that focusing on their symptoms made them more preoccupied with their thoughts, and served as a reminder of past periods of acute illness. For others the questions led to unfavourable comparisons between their desired and ideal mental state, and drew attention to the high frequency of their psychotic symptoms and thoughts about suicide. Some individuals stated that the downbeat and clinical nature of the items made them concentrate on their negative emotions and suggested that more neutral questions should be introduced. However, for a few individuals the increased levels of retrospection also had a positive effect, showing them how much progress they had made since they were last acutely unwell.

‘…yeah it got me digging in stuff cos it started me comparing how I am now to how I used to be. And although I do think I’ve come a long way now. I needed… it was a reminder of what I keep in that bag that I keep in the shed. That’s my daemons that I keep in that bag… And it just got me digging and its not good. That’s why I cancelled it. I found it too personal in the end.’ ID16 (female in her thirties)

‘…but at the same time its also shown me, the device has shown me that I’ve also come a long way a long, long way. So that’s a positive.’ ID16 (female in her thirties)

Overall, participants noted little actual change in levels of psychotic symptoms in response to filling in the questions, but some felt that it might exacerbate levels of paranoia in other, more acute, service users. One individual noted that the assessment procedure had made him more attentive to his perceptual abnormalities, whereas others suggested that they had experienced fleeting suspicious thoughts about where the data was being sent and how it was being used.

‘*I’m not very suspicious generally but I found myself going ‘oh this is bit weird’ so I imagine someone in the throes of it not taking their medication would find it very weird but I can only speak for myself. I didn’t find it too intrusive’ ID3 (male it his twenties)*

Many individuals questioned the validity of the mobile-phone assessment items. Firstly, some participants were unsure about the degree to which their numerical responses accurately captured how they were feeling. Some noted the need for greater contextual understanding and elaboration. For instance, one individual noted that his perceptions of threat were justifiable given his situation (the Manchester football derby; ID10 male in his thirties). Others stated that a lack of visual information (e.g. cleanliness, ability to look after oneself) would make it difficult to judge an individual’s mental state. Secondly, many participants felt that other patients would misreport or lie about their symptoms when using self-report items. Indeed, it was thought that some people may wish to underplay (e.g. when feeling guarded) or overplay (e.g. if histrionic or help-seeking) how they were feeling at certain times. Several individuals felt that when unwell or experiencing more severe symptoms, they would have been unable to accurately relay information about their mental state.

‘When I was feeling unwell I probably would have been too erratic to have filled the questions in properly.’ ID14 (male in his forties)

‘Its just a bit more erm… how would you explain it now… you know… the 1–7 its not, its not… I can’t think of the word to say, like that… It doesn’t really tell you exactly what you’re thinking, you know, you know. Are you feeling sad? So you put like 2, but whats a 2, a little bit? So you know maybe theres a reason you feel sad and you might want to add to that or something a bit more.’ ID10 (male in his thirties)

The acceptability of integrating mobile-phone assessment technology into everyday routine was also often discussed. Generally, participants were unconcerned about completing questions in public or private settings, and believed the devices to be well normalised and non-stigmatising. Although individuals were careful not to divulge information about their mental state, many were happy to share their experiences of using the phone devices with other people in terms of doing a research study or monetary incentives. Disruption to activities by mobile-phone assessment was minimal, although some individuals did report changing their plans to take into account the sampling procedure (e.g. waiting for the alarm to sound before going out; ID19 (male in his forties)). Additionally, some participants noted that the procedure was less compatible with a busy lifestyle and that they would struggle to complete entries when at their most active (e.g. work, gym, shops). A few participants were also concerned that their own phone and smartphone would go missing or be stolen if they took them outside and therefore often left them at home. Whilst a large proportion of the sample noted that the phone alarms had interrupted at least one activity during the two weeks of sampling (e.g. sleeping, making dinner), most stated that they did not consider this to be a problem.

‘It didn’t bother me. I’d just sit there and do ‘em. I was sat out in the sun the other week doing ‘em, sat out in the bright sunlight doing ‘em. It didn’t bother me.’ ID8 (male in his late teens)

### Theme 3: Perceived impact on clinical care

Participants evaluated the technology in terms of the effect it would have on their clinical care. Although individuals generally discussed the impact it would have on their specific situation, a few participants spoke more broadly about the effects it could have on the NHS as a whole. For example, one participant noted that although real-time uploading of data could provide more immediate assistance, there is often a lack of on call psychiatrists who are able to respond to clinical information (ID23; male in his forties). Another individual noted that it might overburden his clinician with additional work (ID1; male in his thirties).

The perceived benefits of the technology were often seen in terms of enabling the clinician to help the patient. Many individuals emphasised the advantages of detailed information in directing care and helping clinicians to gain insights into their mental state. For example, some people noted that it would help their psychiatrist to prescribe the right levels or type of medication. Participants also echoed the researchers’ expectations that real-time data collection could lead to earlier and more effective intervention. This was often seen in terms of managing risk in vulnerable others, and not monitoring for signs of relapse in healthy and stable individuals. Those few individuals who believed that their clinicians were unable to help them, also made more negative appraisals of the mobile phone procedure, suggesting that confidence in clinicians’ ability to treat mental health may incentivise people to comply with mobile-phone interventions.

‘Yeah they can’t really change the way you feel, it’s just like bringing it up again about you’ve been ill so here’s some questions and… but it don’t do nothing for you, it don’t make you, you think oh yeah I did hear voices so I nearly lost the plot y’know what I mean. I don’t know how… how it helps you.’ ID13 (female in thirties)

‘If somebody is in danger then you want to sort it out as quickly as possible don’t you? To save everybody problems later on… so yeah I think it would be important and it would be good that the psychiatrist has that information.’ ID23 (male in his forties)

A further perceived advantage of mobile-phone based mobile-phone assessment was its ability to foster better communication between clinicians and service-users. Some participants expressed difficulties in retrospectively recollecting symptoms at their infrequent meetings with clinical staff. Others stated that they had felt uncomfortable or pressurised when discussing sensitive issues face-to-face, and that they would sometimes prefer to answer questions about their symptoms remotely. The opportunity to vent and communicate one’s thoughts, especially when alone, was also often seen as a major benefit of the mobile-phone assessment technology.

‘I think it’s a good idea really, because I prefer to just put it in to do something like that because I struggle with talking about things anyway, so with that you can say exactly what you think. I know it was limited to a certain amount of questions weren’t it, but at least on there you can just put in whatever you feel and it’s alright really. ‘ ID9 (male in his late teens)

Participants often discussed the threat mobile-phone technology could pose to their existing care. Many participants emphasised the importance of face-to-face contact with their clinical team, and the benefits that this social interaction has had on their mental state. Conversely, the mobile-phone devices were often seen as impersonal and unable to address important everyday practical problems (e.g. financial difficulties, housing). Therefore, although many individuals acknowledged that a greater level of data could be helpful to their clinician, most felt that it is not a substitute for the human element of their care. One individual summarised this by stating that mobile-phone assessment is for clinicians, whereas interviews are for the patient (ID15; male in his twenties).

‘I think you’re probably better off having someone to talk to or, you know if you’ve got health care professionals, that’s like they know how to deal with situations if they arise, don’t they .’ ID1 (male in his thirties)

Many individuals believed that although there were benefits of integrating mobile-phone technology into people’s clinical case management, they did not think it would be relevant to their personal situation. The reasons for this varied. Whilst some individuals thought themselves to no longer be mentally unwell, others felt that their existing contact with services made additional monitoring redundant. Some participants said that during periods of stability in symptoms or remission many of the advantages of mobile-phone assessment would be lost, and that this form of assessment would be ‘overkill’ (ID1; male in his thirties). One participant felt that meeting his psychiatrist gave him more control over his treatment as he was able to emphasise his priorities and needs (ID22; male in his thirties).

‘I think I am probably past the point where it would be most useful for me. If I was just out of hospital and I was still having symptoms, a large part of the day, then I mean, it would be more useful to monitor my reactions and how my mood has been because I used to fluctuate a lot more than I do now. I used to have quite dark periods in the day and sort of…but it would be fluctuating depending on the voices. So I mean if I was just out of hospital I can see it being used to monitor reactions and my mood, but it would be more useful then.’ ID15 (male in his twenties)

## Discussion

The aim of this study was to explore individuals’ understanding and perceptions of mobile-phone based psychotic symptoms monitoring, in order to ascertain how it might be introduced into their everyday lives and clinical care. Three themes were identified in the data: i) the appeal of usability and familiarity, ii) acceptability, validity and integration into domestic routines, and iii) perceived impact on clinical care.

Participants valued the added usability and functionality of the native software application, but also the familiarity and convenience of using a text-message system on their own phones. Thus, the appeal of and ability to establish familiarity are likely to be important elements when implementing mobile-phone assessment in individuals with psychosis, particularly in the face of more technologically advanced software options. Some authors have suggested that becoming an established healthcare technology user initially requires perseverance, adjustment and the overcoming of software defects [26]. It may therefore be important to provide a high level of assistance and encouragement in the early stages of technology integration. As smartphone technology becomes increasingly widespread, software applications may become an attractive option for delivering assessment on patient’s own phones, thereby reducing the need for them to adjust to a new device. In the more immediate future, it may be beneficial to provide patients with a choice of software, in order to accommodate their preferences and individual priorities. Indeed, healthcare professionals have sometimes emphasised the importance of being flexible and adaptable when integrating technology into services and patient care [21]. Choice of technology should be considered in the context of longer entry times, which may lead to greater levels of non-compliance [13].

In general, participants considered the assessment procedure to be well-normalised and effectively integrated into daily routine. However, many participants stated that the repetitiveness of the questions would have disinclined them from completing mobile-phone based assessment over longer periods of time. In the future, it may be useful to adjust sampling rates according to the purpose of the assessment. For example, when assessing short-term medication effects high frequency sampling may be appropriate, whereas relapse monitoring may require less intense sampling rates over longer periods of time. Compliance may also be improved through machine learning in order to modify and continuously adapt questions and sampling rates according to an individual’s previous responses (e.g. present endorsed items more frequently and vice versa), thus reducing burden and increasing the relevancy of the items [29].

In some cases mobile-phone assessment led to a preoccupation with ones thoughts, and comparisons between the individual’s real, desired and past mental states. This could be considered in terms of biographical disruption, where the onset of a chronic disorder is thought to assault individual’s existing self-narrative and perceived social ties, which in turn leads to an ongoing aversive state dominated by attempts to make sense of and manage illness uncertainties [30,31]. Repeated symptom assessments may force participants to constantly re-evaluate how they see themselves in relation to their diagnosis, leading to greater levels of biographical disruption. It may be beneficial for clinicians and clients to reflect on the personal salience of the momentary assessment procedure in order to minimise related distress and the impact it has on their reporting. It has also been proposed that an internal preoccupation can lead to an undesirable state of cognitive dissonance, which can generate socially unacceptable interpretations of one’s own thoughts and psychosis [32].

Participants believed that mobile assessment could benefit care in terms of earlier intervention and more detailed data delivery, which is in contrast to the ambivalence shown by participants in other healthcare technology evaluations [20]. Interestingly, patients also advocated that mobile assessment could be a useful method for improving communication between service users and clinicians, and thus for developing a therapeutic alliance. In this respect, as the technology makes the transition into clinical practice, it will be important to monitor resultant changes in the interpersonal dynamics between patients and healthcare staff [33]. A further benefit of the mobile-phone assessment procedure was the therapeutic effect of being able to vent and relay ones experiences. Thus, the qualitative interviews uncovered unknown utilities of the mobile-phone assessment procedure.

Despite the benefits it could bring to patient-clinician communication, participants’ emphasised the importance of retaining a human element to care, and not relying solely on impersonal and distant computer based assessment. Indeed, social support from one’s clinical team was seen as an important factor in facilitating recovery and mental wellbeing. Participants also highlighted the fallacy of relying solely on patient self-report, and a need for expansion and contextual understanding. The perceived threat to existing care was identified as a reason for non-participation in or withdrawal from one large randomised trial for TeleHealth [22]. Another study found that people are also often sceptical about the viability of using mobile devices as an alternative to in-person service delivery [21]. From a user perspective, mobile-phone based assessment should be introduced as an adjunct to, rather than a substitute for, current clinical practice.

Although the qualitative interviews highlighted benefits of mobile-phone based assessment, in line with other studies of acceptance of telecare [21], many patients did not believe it to be applicable to their specific situation. It is possible that the loss of reality testing and monitoring, and the subsequent reduction in insight, associated with schizophrenia may reduce self-perceived relevance of such technologies into the patients’ lives [34]. Some patients felt that their routine meetings with clinical staff or the stability of their symptoms made additional monitoring redundant. With regards to the latter, mobile-phone assessment might be most effective when a patient is in a state of change or prone to symptom fluctuation (e.g. immediately after being discharged from hospital, when starting a new form of medication). When integrating this technology into clinical practice it will be important to identify mutually understandable benefits of the more detailed, real-time assessment. Low perceived need for intervention has previously been identified as the primary barrier for not help seeking by suicidal individuals [35]. Additionally the separation from reality associated with the emergence of psychotic symptoms may impede the help negotiation process further. Mobile-phone based assessment provides objective checks for patients’ symptomatology assuming they report honestly and openly their subjective experiences. Successful implementation of mobile-phone technology may, therefore, be contingent on a clearly described an understandable purpose or goal and gains for the patient involved [21,25].

Qualitative interviews often utilise smaller sample sizes than would be accepted within quantitative research. However, it does not aim to overstate its findings by generalising results to other populations. Instead, qualitative research seeks to provide description and theories that are relevant to the particular setting [36]. Thus, the findings should be taken with caution, recognising that the study took place with a specific population, and is time and context specific. Whilst considering these limitations it is important to recognise that research has suggested that many themes resulting from qualitative interviews can resonate strongly with findings from comparable research studies [37].

## Conclusion

As mobile-phone based assessment for psychosis makes the transition from research to clinical tool, it is vital that user feedback is gathered and integrated into new iterations of the approach. The feedback presented in this paper suggests that patients are conscious of the benefits that mobile-phone assessment could bring to clinical care, and that it can be successfully integrated into everyday routine, supporting the findings of earlier quantitative analysis [13]. However, it is also clearly important to demonstrate to patients the personal, as well as theoretical, benefit that the technology can bring. The mobile-phone assessment procedure itself may need to be tailored to the individual, potentially through machine learning, in order to maximise compliance.

## Competing interests

No competing interests, financial or otherwise, arise from this research.

## Authors’ contributions

JPC recruited and assessed all participants, analysed the data and co-wrote the manuscript. AR and LL assisted with coding, identification of emerging themes and the writing of the manuscript. JA was project manager, coordinating software design and development. MM created and designed the mobile-phone software, and assisted with the writing up of the findings. CB, TW, SK and EB sat on the steering committee for this project, assisting with its design and commenting on several drafts of this manuscript. SL was the primary investigator and grant holder, who designed and managed the study. All authors read and approved the final manuscript.

## Pre-publication history

The pre-publication history for this paper can be accessed here:

http://www.biomedcentral.com/1471-244X/13/34/prepub
